# One-Step Theory View on Photoelectron Diffraction: Application to Graphene

**DOI:** 10.3390/nano12224040

**Published:** 2022-11-17

**Authors:** Eugene Krasovskii

**Affiliations:** 1 Departamento de Polímeros y Materiales Avanzados, Física, Química y Tecnología, Universidad del Pais Vasco/Euskal Herriko Unibertsitatea, 20080 Donostia/San Sebastián, Basque Country, Spain; eugene.krasovskii@ehu.eus; 2 Donostia International Physics Center (DIPC), 20018 Donostia/San Sebastián, Basque Country, Spain; 3 IKERBASQUE, Basque Foundation for Science, 48013 Bilbao, Basque Country, Spain

**Keywords:** graphene, angle-resolved photoemission, electron scattering, augmented plane waves

## Abstract

Diffraction of photoelectrons emitted from the core 1*s* and valence band of monolayer and bilayer graphene is studied within the one-step theory of photoemission. The energy-dependent angular distribution of the photoelectrons is compared to the simulated electron reflection pattern of a low-energy electron diffraction experiment in the kinetic energy range up to about 55 eV, and the implications for the structure determination are discussed. Constant energy contours due to scattering resonances are well visible in photoelectron diffraction, and their experimental shape is well reproduced. The example of the bilayer graphene is used to reveal the effect of the scattering by the subsurface layer. The photoemission and LEED patterns are shown to contain essentially the same information about the long-range order. The diffraction patterns of C 1s and valence band photoelectrons bear similar anisotropy and are equally suitable for diffraction analysis.

## 1. Introduction

Photoelectron diffraction (PED) [1,2,3] has long been established as a powerful tool for the determination of the surface crystal structure along with the low-energy electron diffraction (LEED) [4,5] and very low-energy (VLEED) I(V) technique [6]. A conclusive interpretation of the diffraction patterns and a reliable determination of the atomic geometry depend on an adequate theoretical modeling of multiple scattering. Powerful computational methods have been developed that treat the multiple scattering in real space and are indispensable for the study of the short-range order of surfaces and adsorbates [7,8,9]. Recent progress in the fabrication of two-dimensional (2D) materials (such as graphene or h-BN) draws attention to methods of determining the geometry of long-range-periodic 2D crystals. The recently developed angle-resolved reflected-electron spectroscopy (ARRES) technique [10,11] has allowed angular scanning of electron reflection at very low energies and revealed rich structure of the $R(E,k_{\left|\right|})$ diffraction patterns carrying information about the surface geometry and the electronic structure. The angular distribution of photoemitted electrons $I(E,k_{\left|\right|})$ presents an alternative diffraction pattern. Currently, the experimental focus has been on ultra-high kinetic energies [3], where the theoretical analysis allows certain simplifications [12]. On the other hand, low energies have some advantages, such as higher sensitivity to the local environment and lesser effect of atomic vibrations. In the present work, the low-energy regime is theoretically explored with the aim to interpret the electron reflection and photoemission maps on the same footing and understand the relation between PED and LEED.

This calls for theoretical methods capable of treating periodic systems with minimum adjustable parameters and providing a straightforward connection to the underlying band structure. The basis for such approaches is the one-step photoemission theory, where the photoemission intensity equals the probability of the transition to the time-reversed LEED state $\Phi_{k_{\left|\right|}}^{*}$ [13,14,15,16,17]. The most common implementations of the one-step theory employ the multiple-scattering formalism within the layer-KKR [17,18] or real-space cluster approach [19]. The present study employs a one-step theory realized through the variational embedding method [20], where the scattering properties of a 2D slab are represented by the band structure of a supercell containing the slab. In some contexts, the term PED implies photoemission from atomic-like core states, so the outgoing wave can be calculated as the scattering of a spherical wave emanating from the atom by the surrounding atoms. In this case, one does not need to construct $\Phi_{k_{\left|\right|}}^{*}$ for each detection angle, which is exploited in the PED theories based on Refs. [7,8].

Nevertheless, the application of the one-step theory is instructive because it uses the same function $\Phi_{k_{\left|\right|}}$ to describe scattering by the crystal potential in the LEED and in the PED experiment and is not limited to atomic-like states. The aim of the present work is to compare the diffraction patterns (energy-dependent angular distributions) observed in VLEED and in PED. Monolayer and bilayer graphene are used as examples. The dependence of the PED diffraction patterns on the initial state will be analyzed and the effect of backscattering will be considered.

## 2. Computational Methodology and Approximations

The LEED wave function $\Phi_{k_{\left|\right|}}\left(r\right)$ is a scattering solution for a plane wave incident from vacuum with the energy *E* and surface parallel wave vector $k_{\left|\right|}$. It is a Bloch function with the crystal momentum $k_{\left|\right|}$, and inside the graphene slab it satisfies the Schrödinger equation with the Hamiltonian $\hat{H}=-\Delta +V\left(r\right)$. In the present calculation, the imaginary potential responsible for the inelastic scattering is not included. The crystal potential V(r) is the self-consistent all-electron Kohn–Sham potential obtained within the local density approximation of the density functional theory using the numerical procedure described in Ref. [21].

**Figure 1:**
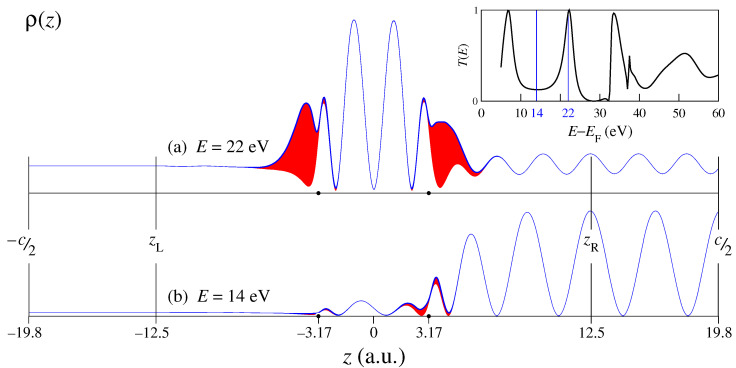
Density profile ρ(z), see Equation (3), of two LEED states at normal incidence at the *AB*-stacked graphene bilayer: (**a**) $E-E_{F}=22$ eV, (**b**) $E-E_{F}=14$ eV. The carbon layers are at z=±3.17 a.u. The shaded areas show the contribution from the $G_{\left|\right|}\neq 0$ surface Fourier harmonics. The inset shows the normal incidence transmission spectrum.

The scattering calculation setup for the graphene bilayer is shown in Figure 1. (The parameters for the monolayer are presented in Ref. [22].) The plane wave normalized to unit flux is incident from the right vacuum half-space $z>z_{R}$, and the current in the left vacuum half-space, $z<z_{L}$, is the transmitted current T(E). In the scattering region $\left[z_{L},z_{R}\right]$ the wave function is a linear combination of the eigenfunctions $\hat{H}\xi_{n}=\epsilon_{n}\xi_{n}$ of an auxiliary three-dimensional *z*-periodic crystal (lattice constant c=39.6 a.u.), which contains the graphene slab as part of the unit cell [20]. The solution of the scattering problem is sought as a linear combination of the basis functions $\xi_{n}$ that satisfies the Schrödinger equation for the energy *E* in the scattering region $z\in [z_{L},z_{R}]$ and at the boundaries $z_{L}$ and $z_{R}$ matches the function and derivative of the plane-wave representations in the respective half-spaces (where it satisfies the Schrödinger equation by construction). The problem is solved with the variational embedding method in the augmented plane waves (APW) representation [20]. The basis set in the scattering region comprises the $\xi_{n}$ functions with energies up to $\epsilon_{\max}$∼130 eV above the Fermi energy, which for the bilayer graphene amounts to around 400 $\xi_{n}$ functions.

To calculate the momentum matrix elements, a Laue representation of the LEED state is constructed by a straightforward expansion of the all-electron wave function in terms of 11,999 plane waves (with wave vectors smaller than 9.8 a.u.${}^{-1}$). It comprises 19 surface reciprocal lattice vectors $G_{\left|\right|}$:

(1) $$\Phi_{k_{\left|\right|}}\left(r_{\left|\right|},z\right)=\sum_{G_{\left|\right|}}\phi_{G_{\left|\right|}}\left(z\right)\exp\left[i\left(k_{\left|\right|}+G_{\left|\right|}\right)\;r_{\left|\right|}\right].$$

The photocurrent $I(E,k_{\left|\right|})$ is calculated as the probability of transition from the eigenstate of the graphene slab $|\;i\;k_{\left|\right|}\;\rangle$ to the time-reversed LEED state $|\;f\;k_{\left|\right|}\;\rangle$:

(2) $$I\left(E,k_{\left|\right|}\right)∼\sqrt{E-E_{\mathrm{vac}}}\;{\left|\langle \;f\;k_{\left|\right|}\;|\hat{o}|\;i\;k_{\left|\right|}\;\rangle \right|}^{2},$$

where $\langle \;r\;|\;f\;k_{\left|\right|}\;\rangle =\Phi_{-k_{\left|\right|}}^{*}\left(r\right)$ and $\hat{o}$ is the dipole operator, $\hat{o}=-i\nabla \cdot e$. The light polarization e in the present calculation is chosen to be along the surface normal. In order to draw the connection with Ref. [23], let us write the Bloch state $\langle \;r\;|\;i\;k_{\left|\right|}\;\rangle$ of an isolated initial-state band λ as a lattice sum of Wannier functions: $\psi_{\lambda k_{\left|\right|}}\left(r\right)=\sum_{R}\exp\left(ik_{\left|\right|}R\right)\eta_{\lambda }\left(r-R\right)$. (The final state cannot be written in this form because $\Phi_{k_{\left|\right|}}$ is not $k_{\left|\right|}$ periodic.) Then it follows immediately from Equation (2) that the transition matrix element is $\int \;\Phi_{-k_{\left|\right|}}\left(r\right)\;\hat{o}\;\eta_{\lambda }\left(r\right)\;dr$, which is physically the same as Equation (5) of Ref. [23]. Note that this expression is not a result of a Brillouin zone averaging. For the emission from core states, the Wannier function is clearly close to a linear combination of the atomic-like states, while for the valence band $\eta_{\lambda }\left(r\right)$ may have a complicated shape and be rather extended.

## 3. Structure of the LEED Wave Functions

Two examples of scattering solutions for the *AB*-stacked bilayer graphene for the normal incidence ($k_{\left|\right|}=0$) are presented in Figure 1a,b for E=22 and 14 eV, respectively, as the density profiles

(3) $$\rho \left(z\right)=\int |\;\Phi_{k_{\left|\right|}}\left(r_{\left|\right|},z\right){\;|}^{2}\;dr_{\left|\right|}.$$

The energy E=14 eV lies at the lowest transmission minimum, while E=22 eV is close to a transmission resonance T(E)=1 [10,11,24], see the inset in Figure 1. With increasing the number of layers, the interval around the T(E) minimum becomes a $k_{\left|\right|}$-projected gap, while the number of the T(E) resonances grows, and in graphite they merge to form a conducting band, see Ref. [25]. At the T(E) minimum, the wave function is seen to rapidly decay into the interior of the bilayer, while at the (almost) full transmission the density distribution is almost symmetric relative to the z=0 plane. The symmetric ρ(z) at T=1 is the consequence of the fact that in the absence of reflection, the scattering states for the wave incident from the right and from the left are related by the time reversal.

The red-shaded areas in Figure 1a,b show the contribution of $G_{\left|\right|}\neq 0$ harmonics (surface Umklapps) to the $\Phi_{k_{\left|\right|}}$ function, see Equation (1). Somewhat surprisingly, this contribution is much smaller at the high reflection (which implies strong scattering) than at the transmission resonance. This means that while the scattering resonances can be qualitatively reproduced in a 1D model crystal, the 1D wave function would be rather unrealistic, see also the analysis in Ref. [26].

## 4. Results and Discussion

**Figure 2:**
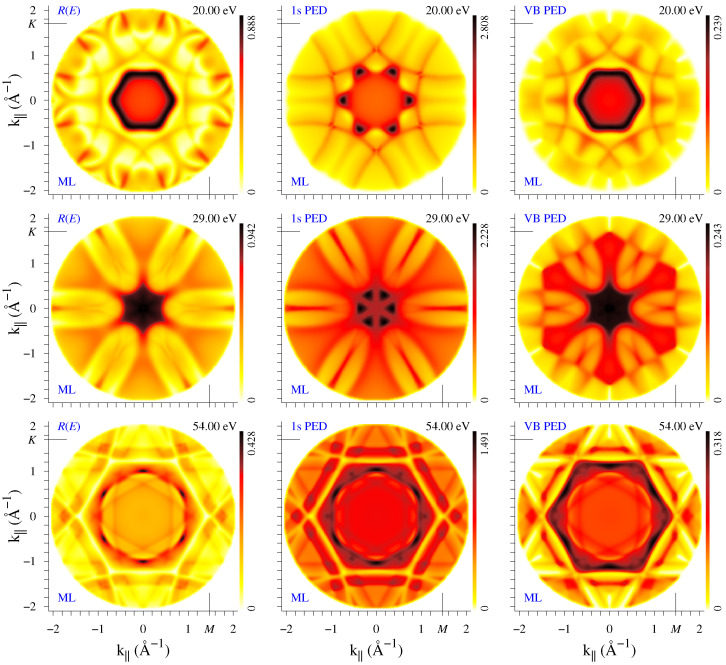
Electron diffraction from the monolayer graphene. Constant energy $k_{\left|\right|}$ distribution of the specular reflectivity *R*, photocurrent from the C 1*s* core states $I_{1s}$, and from the highly dispersive lowermost band $I_{\mathrm{VB}}$ are shown in the 1st, 2nd, and 3rd columns, respectively. Three energies are presented: 20, 29, and 54 eV in the 1st, 2nd, and 3rd rows, respectively. The ΓM line is placed horizontally. The value at the top of the color scale bar indicates the map maximum, which presents the fraction of the specularly reflected current in the *R* maps, and arbitrary units in the *I* maps (the same units in all *I* maps). The full movie for E=5 to 59 eV can be found at film1-MLRCV.mov.

The $k_{\left|\right|}$ distribution of the specular reflectivity *R* for the monolayer graphene is compared in Figure 2 to the photoemission intensity from the 1*s* carbon core band and from the 7 eV-wide lowermost valence band (VB) (see, e.g., Figure 1 in Ref. [27]) for three final-state energies: E=20, 29, and 54 eV. The VB disperses from −19.3 eV at Γ to −12.4 eV at *K*, so the intensity distribution $I_{\mathrm{VB}}\left(k_{\left|\right|}\right)$ implies a scan over the initial state energies. Note that to facilitate the comparison, constant-*E* maps are shown for VB PED rather than constant-ℏω ones.

The three maps in each row manifest similar gross features, which reflect the structure of the $\Phi_{k_{\left|\right|}}$ states, although the details may be rather different. For example, for E=20 eV, the central hexagon of side 0.7 Å${}^{-1}$ has very similar shape in $R(k_{\left|\right|})$ and $I_{\mathrm{VB}}\left(k_{\left|\right|}\right)$, but it looks very differently in $I_{1s}\left(k_{\left|\right|}\right)$. This hexagon originates from a sharp scattering resonance that is specific to graphene: it was theoretically predicted in Ref. [27] and first experimentally observed in Ref. [28]. The present results agree well with the measurements in Ref. [28], regarding both the orientation and dispersion of the hexagon: around 20 eV, its size is about 7% smaller in the present theory than in the experiment (according to the low-transmission signature of the resonance), which corresponds to an upward shift by about 1 eV of the measured unoccupied band structure relative to the calculated one. (This is a typical value of the self-energy correction for the Kohn–Sham states at such energies.) The concave-sided larger hexagon formed by arc-shaped narrow stripes is present in all three E=20 eV spectra, as well as in the measurements of Ref. [28]. At E=29 eV, the small hexagon transforms into a star with rays in the ΓK direction, which again is similar in the *R* and $I_{\mathrm{VB}}$ maps but appears different in the $I_{1s}$ map. On the other hand, the elongated petals pointing in the ΓM direction bear the same shape in all three maps. Apart from this, the VB PED map manifests a hexagonal shape that coincides with the 2D Brillouin zone (BZ) of graphene. The well-visible BZ contour is due to the highly dispersive initial states, whereby the initial state rapidly changes character in crossing the BZ boundary. This feature persists over an energy interval from 20 to 39 eV, see the movie film1-MLRCV.mov provided in the Supplementary Material. Clearly, it provides the most direct information about the lattice constant. Finally, the E=54 eV maps present an opposite example, where the reflectivity and 1*s* emission produce very similar patterns, and the VB one is different.

**Figure 3:**
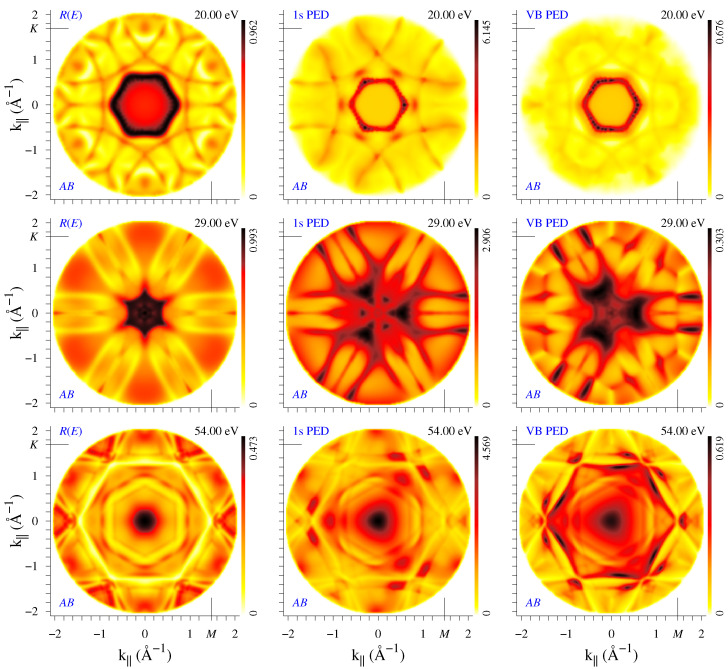
Electron diffraction from the bilayer graphene (*AB* stacking), see the caption of Figure 2. The full movie for E=5 to 59 eV can be found at film2-ABRCV.mov for *AB* stacking and at film5-TWORC.mov for *AA* stacking.

The question of interpreting the PED patterns obtained from an extended initial-state band has been first raised in Ref. [29], where a strong similarity in the anisotropy of the core and VB X-ray emission from Al(100) was observed. A strong PED effect was observed in the ultraviolet emission from Cu(100) and Cu(111) [23], with a significant ℏω dependence. An instructive aspect of the present calculation is that no band-averaging is involved as the extended initial-state band is strictly two-dimensional and energetically isolated. It can be concluded that the three diffraction experiments manifest similar anisotropy, see Figure 2 and Figure 3. For a reliable determination of structural parameters, it may be useful to compare diffraction maps of different incident wave sources in order to reveal common features.

The electron diffraction maps from the *AB* bilayer are presented in Figure 3 for the same three energies. The bilayer bears $C_{3v}$ symmetry, and thus the PED maps naturally do not exhibit vertical-mirror symmetry axis. At the same time, the $R(k_{\left|\right|})$ maps show $C_{6v}$ symmetry, which is the consequence of 3D inversion symmetry of the bilayer. Indeed, one can prove (see Appendix A) that the transmission and reflection coefficients, *t* and *r*, for the incident plane waves with opposite $k_{\left|\right|}$ are related as $r_{-}=r_{+}$ and $t_{-}=-t_{+}^{*}\frac{r_{+}}{r_{+}^{*}}$. Thus, the reflectivity $R={\left|r\right|}^{2}$ is the same for $k_{\left|\right|}$ and $-k_{\left|\right|}$, whereas the wave functions are generally different. Similar to the monolayer graphene, the map at 20 eV is dominated by the scattering-resonance hexagon, which is to be expected since this resonance originates from coupling of the surface-perpendicular and in-plane motion within one layer [27]. Generally, the large-scale features in the monolayer maps have their counterparts in the bilayer maps, but at smaller $k_{\left|\right|}$ the differences are significant, which points to a more important role of the interlayer scattering at smaller incidence (emission) angles.

**Figure 4:**
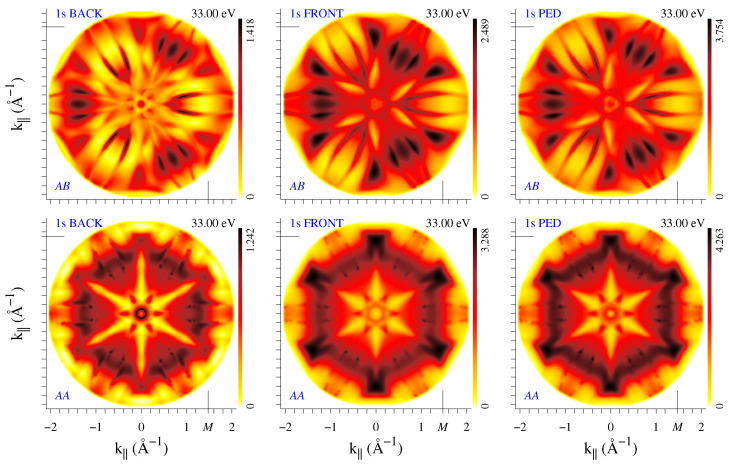
Photoelectron diffraction at 33 eV from the bilayer graphene with * AB* (**upper row**) and *AA* stacking (**lower row**). Left to right: initial 1*s* states at the back layer, at the front layer, and the true full-bilayer states. Full movies are at film3-ABBFT.mov for *AB* and film4-AABFT.mov for *AA* stacking.

**Figure A1:**
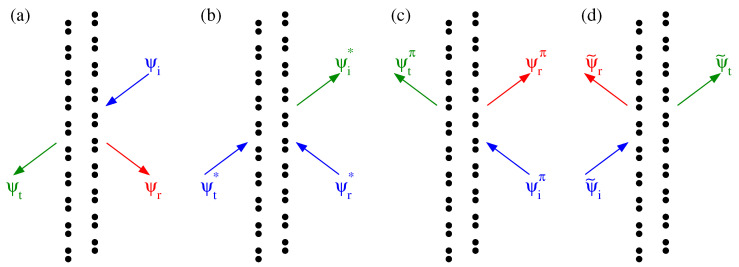
Electron-scattering from an inversion-symmetric bilayer. (**a**) Scattering wave function at some $k_{\left|\right|}$: incident $\psi_{i}$, reflected $\psi_{r}$, and transmitted wave $\psi_{t}$. (**b**) Time-reversed wave function. (**c**) Scattering wave function at $-k_{\left|\right|}$: the electron source of graph (**a**) is rotated by π around the surface normal. (**d**) Spatially inverted wave function.

Let us now consider the role of backscattering in the bilayer graphene. One should keep in mind that the time-reversed LEED state is not the true final state of the photoemission process (as seen from the presence of the incoming wave $\psi_{r}^{*}$ traveling *from* the detector in Figure A1b), so it does not provide detailed information about the multiple scattering of the photoexcited wave. However, for the C 1*s* states, we can make use of the fact that the coherence of the 1*s* orbitals at different sites is physically unimportant and consider separately the emission from the front layer and from the back layer, see Figure 4. In terms of multiple scattering, the electrons photoemitted from the front layer experience extra backscattering, while those emitted from the back layer are additionally scattered by the front layer. For all energies considered, the intensity distribution of the front-layer emission is rather close to the true full bilayer pattern, see Figure 4, while for the back-layer emission this depends on energy (see the full movies at film3-ABBFT.mov for *AB* and film4-AABFT.mov for *AA* stacking). In the example shown in Figure 4, the $I_{1s}\left(k_{\left|\right|}\right)$ patterns from the back and front layers are surprisingly similar.

**Figure 5:**
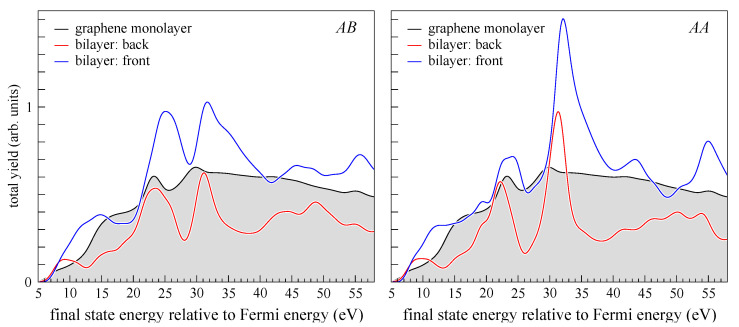
Photoyield from the graphene monolayer (black line with shaded area), the back (red), and front (blue) layer of the bilayer. (**Left**): *AB* stacking, (**right**): *AA* stacking. Shown is the $k_{\left|\right|}$-integrated intensity over the circle of 2 Å${}^{-1}$ radius.

The total photoyield from the front layer is everywhere significantly larger than from the back layer, see Figure 5. This result is in accord with an intuitive single-scattering picture: the photoelectrons emitted from the front layer in the direction away from the detector are reflected from the back layer to add to those traveling towards the detector without backscattering, and the photoelectrons emitted from the back layer are partially reflected from the front layer and do not reach the detector. Indeed, the photoyield from the stand-alone graphene monolayer is larger almost everywhere than from the back layer and smaller than from the front layer for both stackings, see Figure 5.

**Figure 6:**
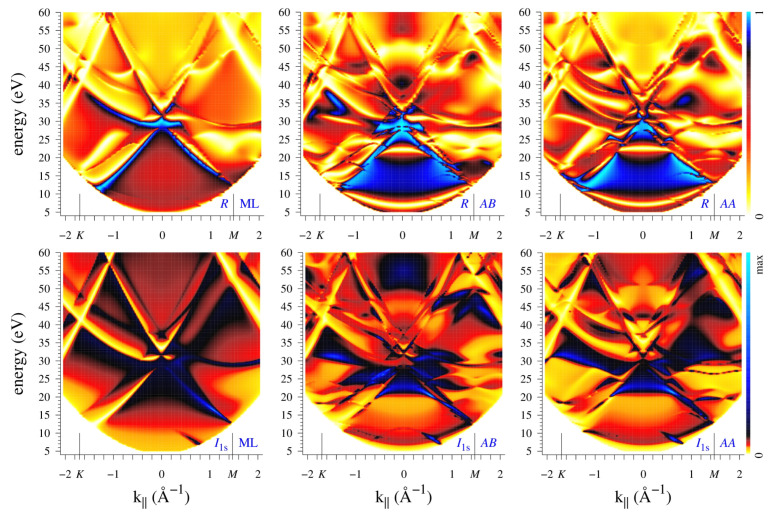
Energy–momentum distribution of reflectivity (**upper row**) and C 1s photoemission (**lower row**) for graphene monolayer and bilayers of *AB* and *AA* stacking, 1st, 2nd, and 3rd columns, respectively. Shown are ΓK (negative $k_{\left|\right|}$) and ΓM (positive $k_{\left|\right|}$) directions.

Diffraction patterns are seen to depend substantially on the number of layers and on the stacking order, concerning not only the higher symmetry of the $I_{1s}\left(k_{\left|\right|}\right)$ distribution for *AA* stacking but often the overall shape of both the $I_{1s}\left(k_{\left|\right|}\right)$ and $R(k_{\left|\right|})$ patterns (as in the example of Figure 4). However, at certain energies there appear thin arc-like stripes that are present both in $I_{1s}\left(k_{\left|\right|}\right)$ and $R(k_{\left|\right|})$ maps in all the systems, for example, in Figure 2 and Figure 3 one can see the lines of enhanced (dark lines at 20 eV) or reduced (light lines at 54 eV) reflectivity. The light lines form the transmission slits typical of 2D crystals. They are best seen in the $R(k_{\left|\right|},E)$ plots in the upper row of Figure 6. For both bilayers, two transmission slits due to interlayer scattering appear around Γ at low energies: the large concave-up arc (well-known experimentally [10,11,24]) disperses from 6 to 12 eV and the small concave-down arc from 21 to 23 eV. Both transmission resonances correspond to an enhanced photoemission intensity, see lower row in Figure 6. Apart from this, in both directions, $R(k_{\left|\right|},E)$ manifests almost straight high-transparency lines with negative dispersion: from 55 eV at 1.2 Å${}^{-1}$ (1.4 Å${}^{-1}$) to 31 eV (43 eV) at 2 Å${}^{-1}$ along ΓK (ΓM). This feature is present both in the bilayers and in the monolayer, which points to its in-layer-scattering origin. In the $I_{1s}\left(k_{\left|\right|},E\right)$ maps, this corresponds to a reduced intensity, in contrast to the features of interlayer origin. Interestingly, in the $I_{1s}\left(k_{\left|\right|},E\right)$ maps it is seen more distinctly than in the $R(k_{\left|\right|},E)$ ones.

We have seen that the difference in stacking results in quite a different structure of the PED and reflectivity maps. However, they rapidly vary with energy, which implies certain difficulties in using them as structure fingerprints. At the same time, in the present example there exist gross features that clearly discriminate between *AB* and *AA* stacking: Note the rapid growth in reflectivity above 50 eV around the normal incidence for *AB* stacking, which does not occur for the *AA* stacking. It is accompanied by an even stronger increase in C 1s emission from the *AB* bilayer that is not seen in the *AA* one. (Interestingly, for $k_{\left|\right|}=0$, both R(E) and $I_{1s}\left(E\right)$ spectra are virtually identical for the two stackings below 30 eV.) To summarize, the scattering of the incoming (LEED) and outgoing (PED) electrons share many common features; however, the respective structures may bear the same or inverse (maxima instead of minima) character.

## 5. Conclusions

The present proof-of-concept calculation demonstrates the prospect of employing the PED technique at low energies in application to 2D crystals and interpreting energy-dependent intensity maps within the one-step photoemission theory. In particular, in the example of graphene monolayer and bilayers, the LEED and PED patterns are shown to contain essentially the same information about the long-range order, the main difference being that for the *AB*-stacked bilayer, the LEED pattern bears higher symmetry than the PED pattern (owing to the combination of time-reversal and inversion symmetry). The calculations confirm the important role of in-layer scattering in graphene multilayers: many characteristic features are already present in the graphene monolayer. The layer-resolved C 1s PED patterns are found to be sensitive to the location of the initial state, but this depends on the final-state energy. On average, the photoemission from the back layer is weaker than from the front layer, which is the result of solely elastic scattering. The PED patterns from the atomic C 1s and extended VB states differ in detail but have similar anisotropy and common large-scale features and are equally suitable for diffraction analysis.

## Data Availability

Not applicable.

## Supplementary Materials

The following supporting information can be downloaded at: https://www.mdpi.com/article/10.3390/nano12224040/s1, Video S1: film1-MLRCV.mov. Video S2: film2-ABRCV.mov. Video S3: film3-ABBFT.mov. Video S4: film4-AABFT.mov. Video S5: film5-TWORC.mov.

## Appendix A. Proof that for a Centrosymmetric Slab It Is ***R*(**-k**_||_**,***E*) = *R*(**k**_||_**,***E*)**

Consider the incident $\psi_{i}$, reflected $\psi_{r}$, and transmitted wave $\psi_{t}$ in Figure A1a,

(A1) $$\begin{array}{cc} \psi_{i}\left(r\right) & =\;\;\exp i(k_{\left|\right|}r_{\left|\right|}+k_{⊥}z), \end{array}$$

(A2) $$\begin{array}{cc} \psi_{r}\left(r\right) & =r\exp i(k_{\left|\right|}r_{\left|\right|}-k_{⊥}z), \end{array}$$

(A3) $$\begin{array}{cc} \psi_{t}\left(r\right) & =t\exp i(k_{\left|\right|}r_{\left|\right|}+k_{⊥}z). \end{array}$$

Figure A1b shows the time-reversed wave function and Figure A1c the scattering of the wave with opposite momentum $-k_{\left|\right|}$ (the electron source is rotated by π around the surface normal). Finally, Figure A1d is the spatial inversion of Figure A1a: $\tilde{\psi }\left(r\right)=\psi \left(-r\right)$. We are interested in the reflection and transmission coefficients for the case “c”:

(A4) $$\begin{array}{cc} \psi_{r}^{\pi }\left(r\right) & =r^{\pi }\exp i\left(-k_{\left|\right|}r_{\left|\right|}-k_{⊥}z\right), \end{array}$$

(A5) $$\begin{array}{cc} \psi_{t}^{\pi }\left(r\right) & =t^{\pi }\exp i\left(-k_{\left|\right|}r_{\left|\right|}+k_{⊥}z\right). \end{array}$$

Case “d” can be represented as a linear combination of cases “b” and “c”: $\tilde{\psi }=b\psi^{*}+c\psi^{\pi }$. By equating the coefficients of each of the four waves in both half-spaces, we can write:

(A6) $$\begin{array}{cccc} \exp i(-k_{\left|\right|}r_{\left|\right|}-k_{⊥}z)= & \;bt^{*}\exp i\left(-k_{\left|\right|}r_{\left|\right|}-k_{⊥}z\right) & \; & \;\mathrm{incident}\;\mathrm{from}\;\mathrm{the}\;\mathrm{left}\;\mathrm{half}-\mathrm{space}, \end{array}$$

(A7) $$\begin{array}{cccc} br^{*}\exp i\left(-k_{\left|\right|}r_{\left|\right|}+k_{⊥}z\right)= & -c\exp i(-k_{\left|\right|}r_{\left|\right|}+k_{⊥}z) & \; & \;\mathrm{incident}\;\mathrm{from}\;\mathrm{the}\;\mathrm{right}\;\mathrm{half}-\mathrm{space}, \end{array}$$

(A8) $$\begin{array}{cccc} \left(b+cr^{\pi }\right)\exp i\left(-k_{\left|\right|}r_{\left|\right|}-k_{⊥}z\right)= & \;\;t\exp i(-k_{\left|\right|}r_{\left|\right|}-k_{⊥}z) & \; & \;\mathrm{outgoing}\;\mathrm{in}\;\mathrm{the}\;\mathrm{right}\;\mathrm{half}-\mathrm{space}, \end{array}$$

(A9) $$\begin{array}{cccc} ct^{\pi }\exp i\left(-k_{\left|\right|}r_{\left|\right|}+k_{⊥}z\right)= & \;\;r\exp i(-k_{\left|\right|}r_{\left|\right|}+k_{⊥}z) & \; & \;\mathrm{outgoing}\;\mathrm{in}\;\mathrm{the}\;\mathrm{left}\;\mathrm{half}-\mathrm{space}. \end{array}$$

This gives $r^{\pi }=r$ and $t^{\pi }=-t^{*}\frac{r}{r^{*}}$, which explains the $C_{6v}$ symmetry of the reflectivity patterns in Figure 3.
